# Supplementary material for: Machine learning reveals sequence and methylation determinants of SaCas9–PAM interactions in bacteria
Source: Nucleic Acids Res. 2026 Jan 15;54(2):gkaf1520. doi: 10.1093/nar/gkaf1520 (PMC12805903; doi:10.1093/nar/gkaf1520)
Supplement: gkaf1520_Supplemental_Files [file gkaf1520_supplemental_files.zip › Ham_Browne_supplement_Dec15.pdf]

Supplementary Information

Machine learning reveals sequence and methylation determinants of SaCas9–PAM interactions in bacteria

Dalton T. Ham[1,2], Tyler S. Browne[1,2], Claire Q. Zhang[1], Gary W. Foo[1], Gregory B. Gloor[1,*], and David R. Edgell[1,3,*]

[1]Department of Biochemistry, Schulich School of Medicine & Dentistry, Western University, London, ON N6A 5C1, Canada
[2]These authors contributed equally
[3]Lead contact
[*]Correspondence: dedgell@uwo.ca, ggloor@uwo.ca

Supplemental information index

- Figure S1. Representative gel images of SaCas9 protein purification
- Figure S2. Schematic of the crisprHAL machine learning architecture
- Figure S3. Plot of sgRNA abundance versus activity for the pTox-KatG enrichmenet and *C. rodentium* depletion experiments
- Figure S4. Plot of T preference at PAM position 6 and [+1] nucleotide
- Figure S5. Summary plots for crisprHAL model performance on different subsets of testing data
- Figure S6. Plots of nucleotide and di-nucleotide preference across (Tev)SaCas9 target sites in pTox-KatG
- Figure S7. Plot of sgRNA activity for sites with single adenine or cytosine methylation in the crRNA region or PAM region in *C. rodentium*
- Figure S8. SaCas9 cleavage of synthetic substrates with 5mC
- Table S1. List of oligonucleotides used in this study
- Table S2. List of sgRNA target sites in pTox-KatG and *C. rodentium* [1]
- Table S3. Summary of ALDEx2 outputs for sgRNA activity against pTox-KatG and *C. rodentium* [1]
- Table S4. Model training and test datasets [1]
- Table S5. Summary of Oxford Nanopore sequencing for *C. rodentium* [1]
- Table S6. Summary table of k_{obs} rates for *in vitro* cleavage of SaCas9/sgRNA targets on pTox-KatG
- Data S1. GenBank file of pTox+KatG [1]
- Data S2. GenBank file of pEndo-TevSaCas9 [1]

[1]File uploaded separately

- Data S3. GenBank file of pEndo-SaCas9 [1]
- Data S4. Fasta file of *C. rodentium* contig [1]

Supplementary Figures

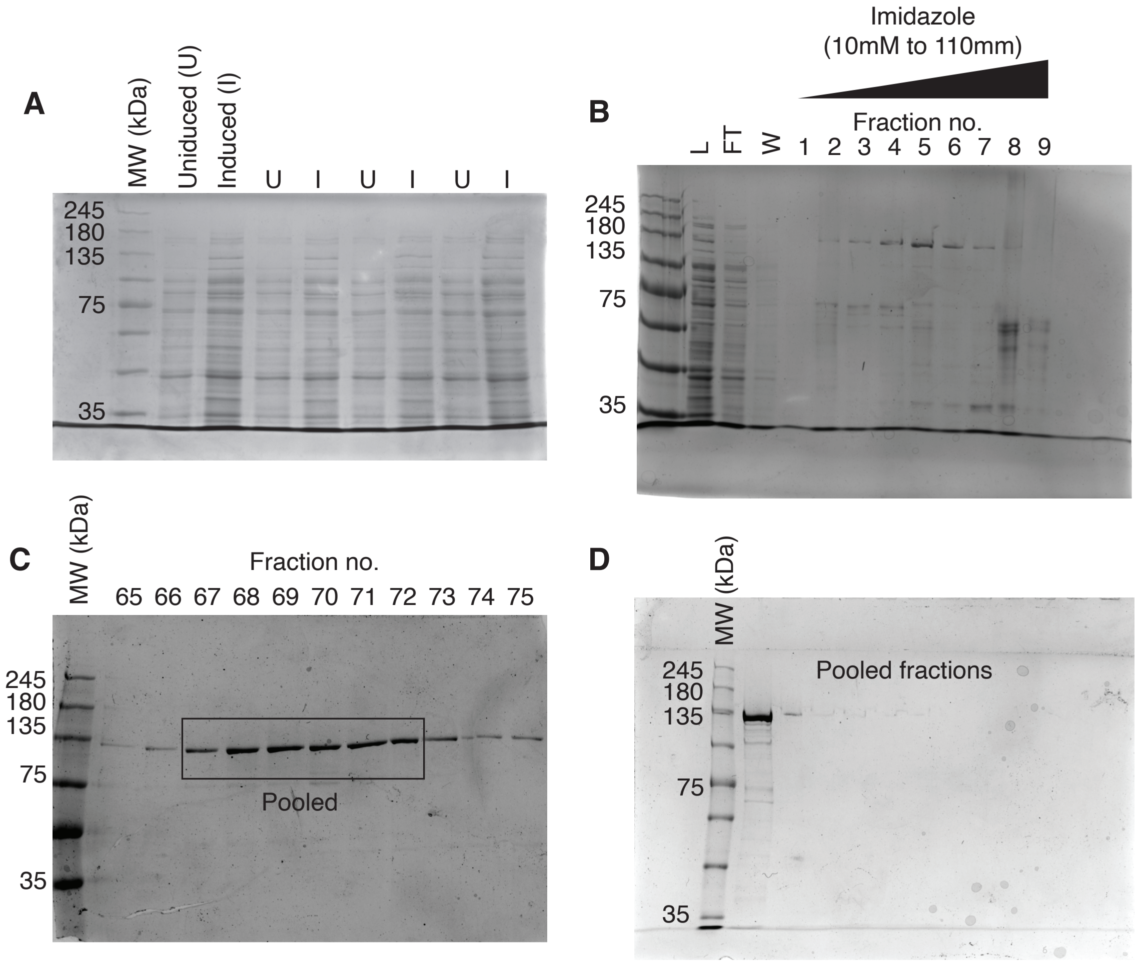

Figure S1: Representative purification of SaCas9 overexpressed in *E. coli*. (A) Gel of total cells from uninduced or induced cultures after overnight growth at 16°. (B) Purification over a HisTrap HP column, with elution fractions labeled and imidazole gradient indicated. L, crude lysate. FT, flow-through from column load. W, wash step. (C) Superdex 200 gel filtration step. (D) Pooled fractions from gel filtration after concentration. In all cases, samples were run on a 12% SDS-PAGE gel alongside the molecular weight marker with sized indicated.

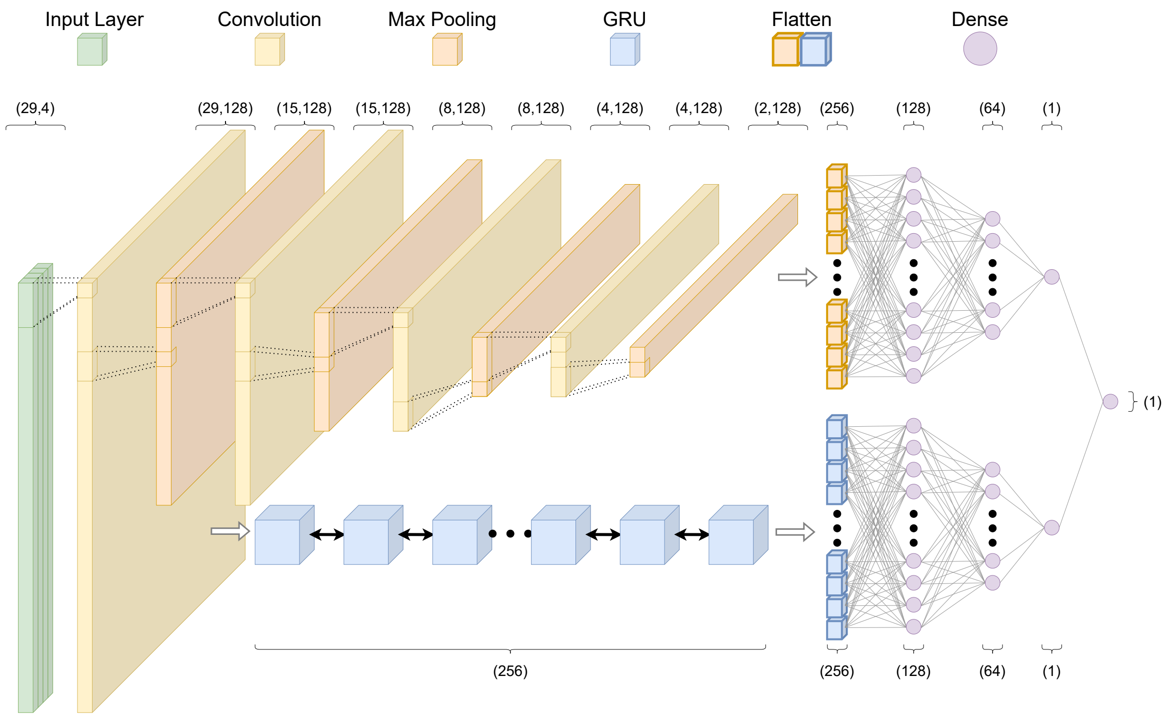

Figure S2: crisprHAL model architecture. A one-hot encoded input nucleotide sequence (input layer, green box) is passed through the dual branch CNN and bi-directional GRU RNN structure, each with subsequent dense layers, resulting in a final output prediction of on-target activity.

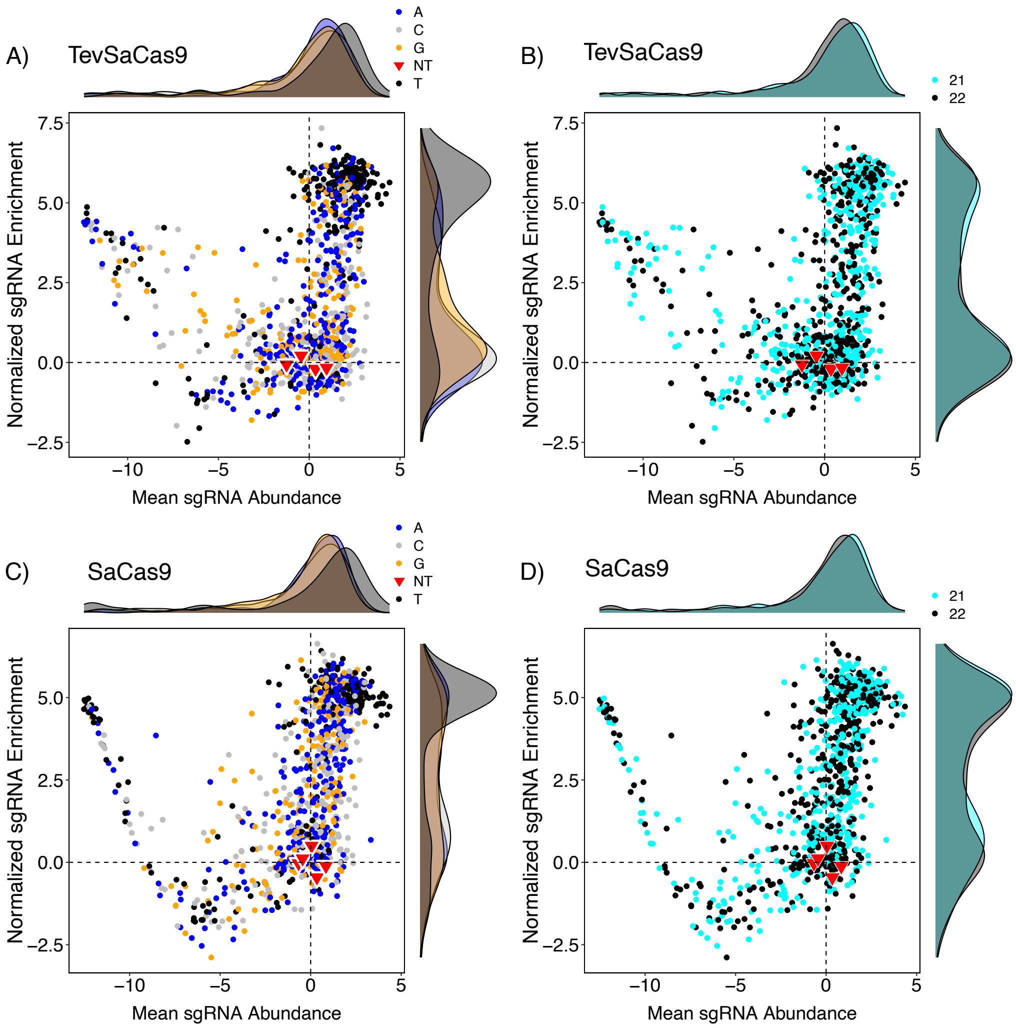

Figure S3: Enrichment experiment for the (Tev)SaCas9/sgRNA pool targeting pTox-KatG. Shown are the plots of normalized sgRNA enrichment versus mean sgRNA abundances for a pool targeting pTox+KatG with TevSaCas9 **(A,B)** and SaCas9 **(C,D)**. In panels **(A and C)** The sgRNAs were separated according to the last nucleotide in 5′-NNGRRN-3′ PAM sequence: C (grey), A (blue), T (black), G (orange) and non-targeting (red). In panels **(B and D)**, the sgRNA are separated by length: 21 nt (cyan), 22 nt (black), and non-targeting (red).

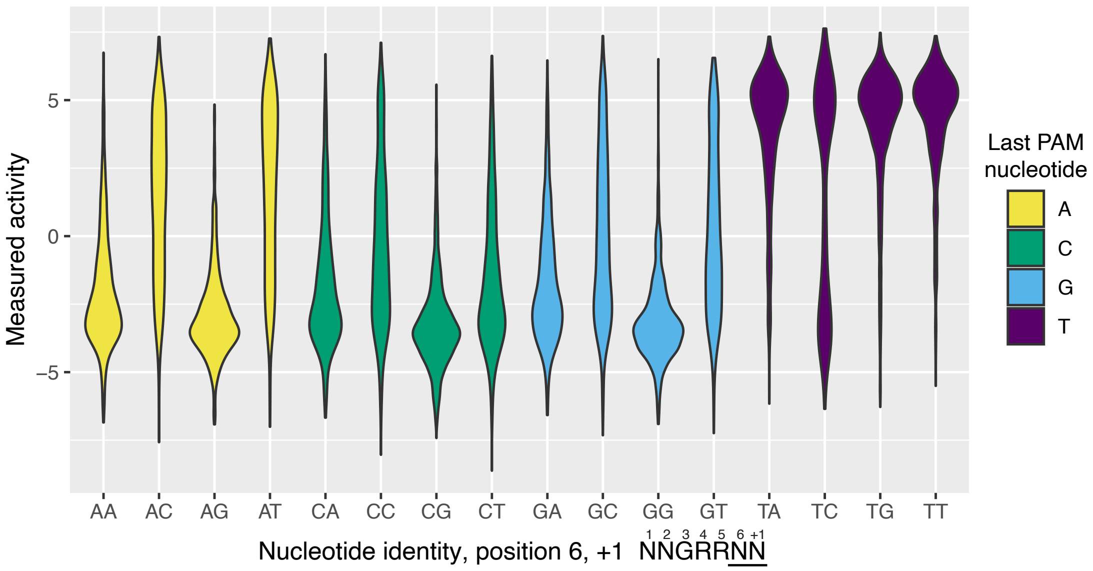

Figure S4: Violin plots of SaCas9 nucleotide preference in PAM position 6 and [+1] flanking nucleotide, colored according to last nucleotide of the PAM sequence at position 6 and grouped by dinucleotide at position 6 and the [+1] position.

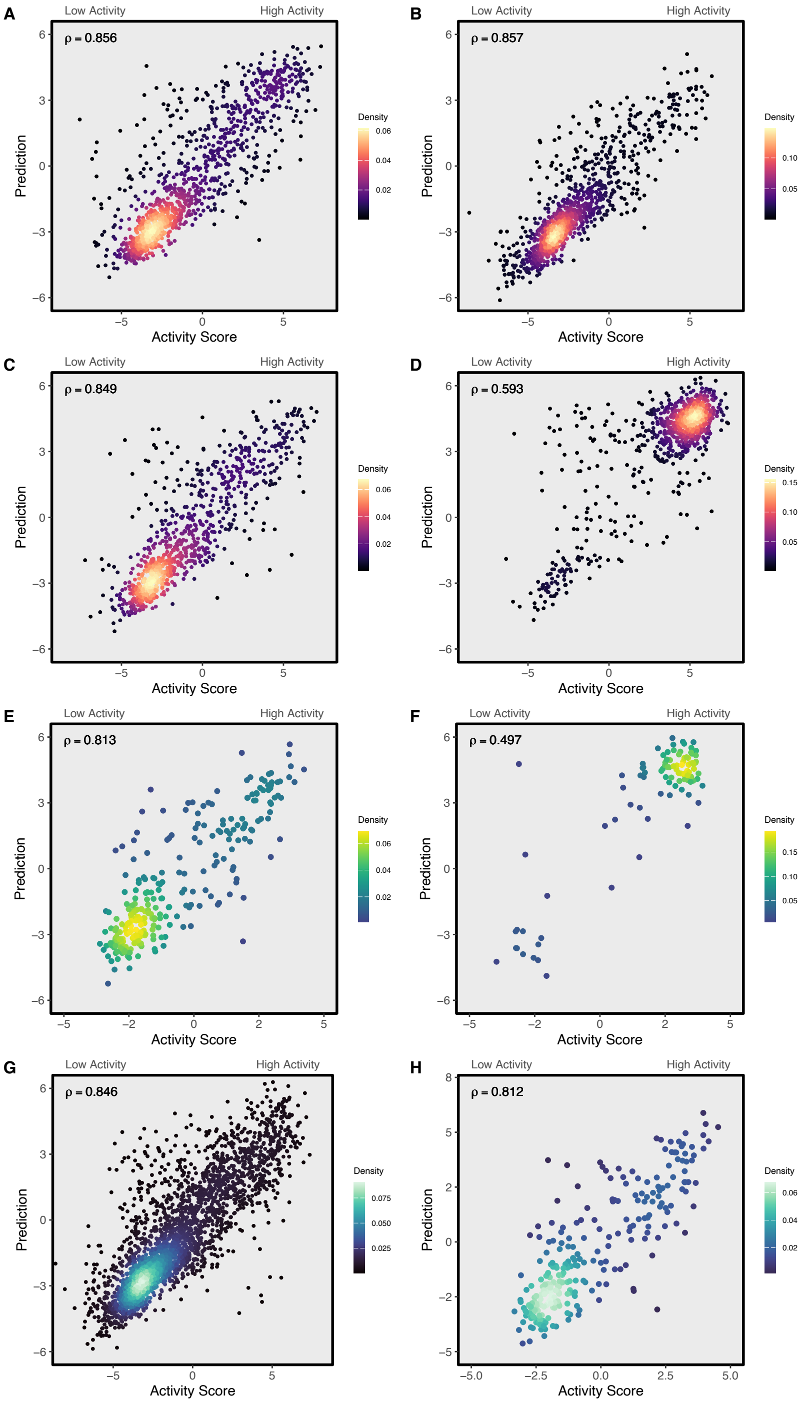

Figure S5: Predictions and Spearman correlations from the final SaCas9 crisprHAL model on subsets of the *C. rodentium* test set for sites with the following PAM sequences (A) NNGRRA, (B) NNGRRC, (C) NNGRRG, and (D) NNGRRT; and subsets of the pTox-KatG test set with the PAM sequences (E) NNGRRV and (F) NNGRRT. Predictions and Spearman correlations from the NNGRRV PAM SaCas9 crisprHAL model on NNGRRV subsets of the (G) *C. rodentium* test set and (H) pTox-KatG test set.

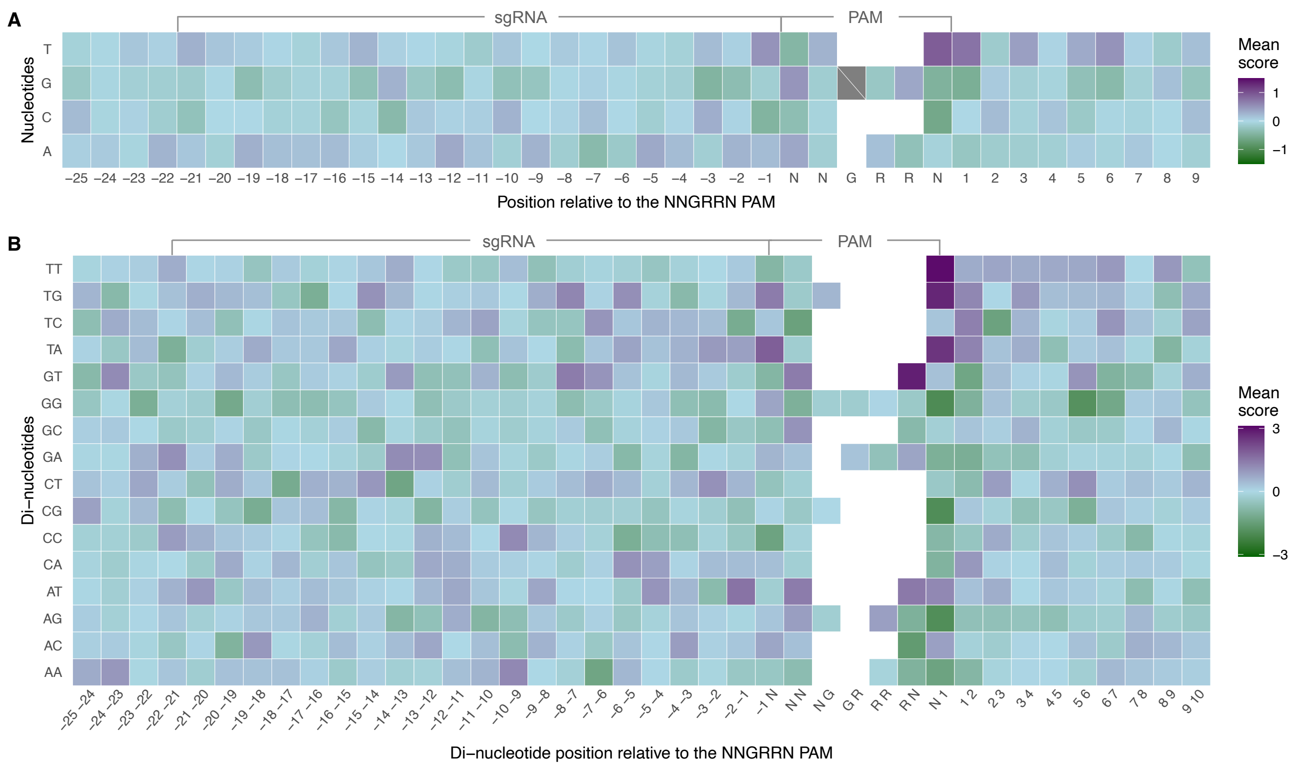

Figure S6: Nucleotide preference across all (Tev)SaCas9/sgRNA target sites in the pTox-KatG enrichment experiment. **(A)** Heatmap of mean single or **(B)** di-nucleotide activity score per position.

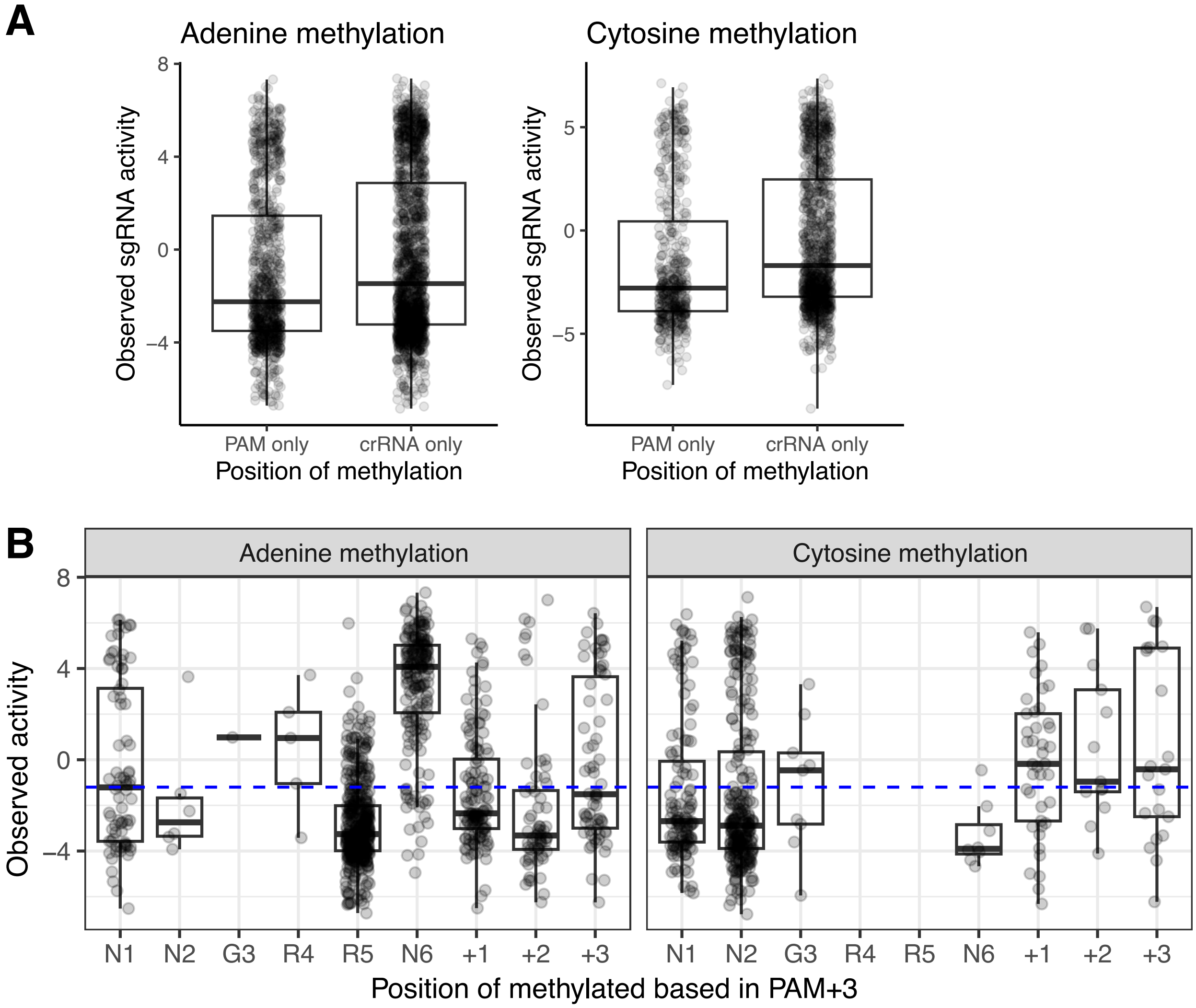

Figure S7: Impact of the position of single adenine and cytosine methylation on sgRNA activity in *C. rodentium*. **(A)** Boxplots of sgRNA activity for single adenine (left) and cytosine (right) methylation events in the PAM region or in the crRNA region. Each point represents the observed activity of a sgRNA targeting a site with the indicated methylation. **(B)** Observed sgRNA activity for adenine or cytosine methylation events mapped to the PAM[+3] region of C. rodentium target sites. Each point represents the activity of an sgRNA with a methylation event at the indicated position. The blue dashed line is the mean observed activity for all sgRNAs tested.

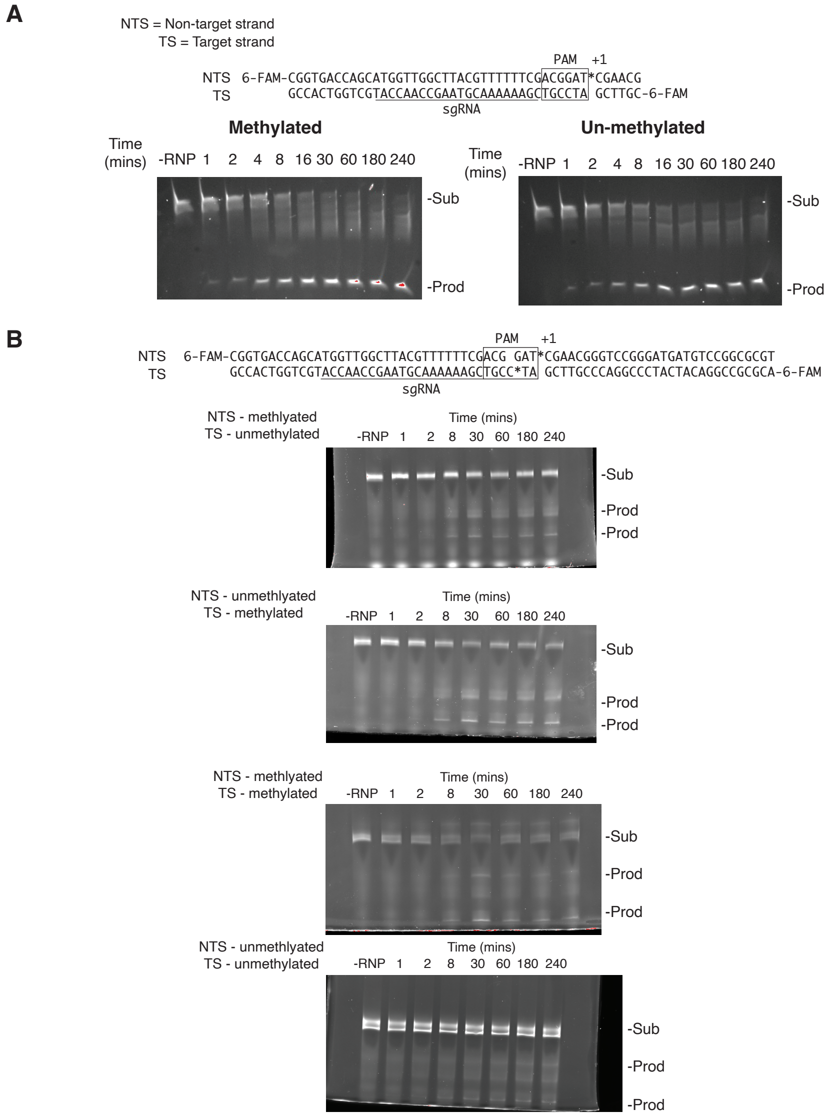

Figure S8: Cytosine (m5C) methylation in the PAM[+1] position does not impact SaCas9 activity on a synthetic DNA substrate. (A) Sequence of target site with 6-FAM modifications at the 5′ ends. Bottom, cleavage time course assays with an RNP consisting of purified SaCas9 and *in vitro* synthesized sgRNAs, or with no added RNP, on the methylated or non-methylated target sites. Aliquots of the stopped reaction were run on a 20% denaturing polyarcylamide gel and visualized on a BioRad Imaging system. The unreacted substrate and products are labeled. (B) Cleavage reactions with a longer synthetic substrate with cytosine methylation in the indicated positions.

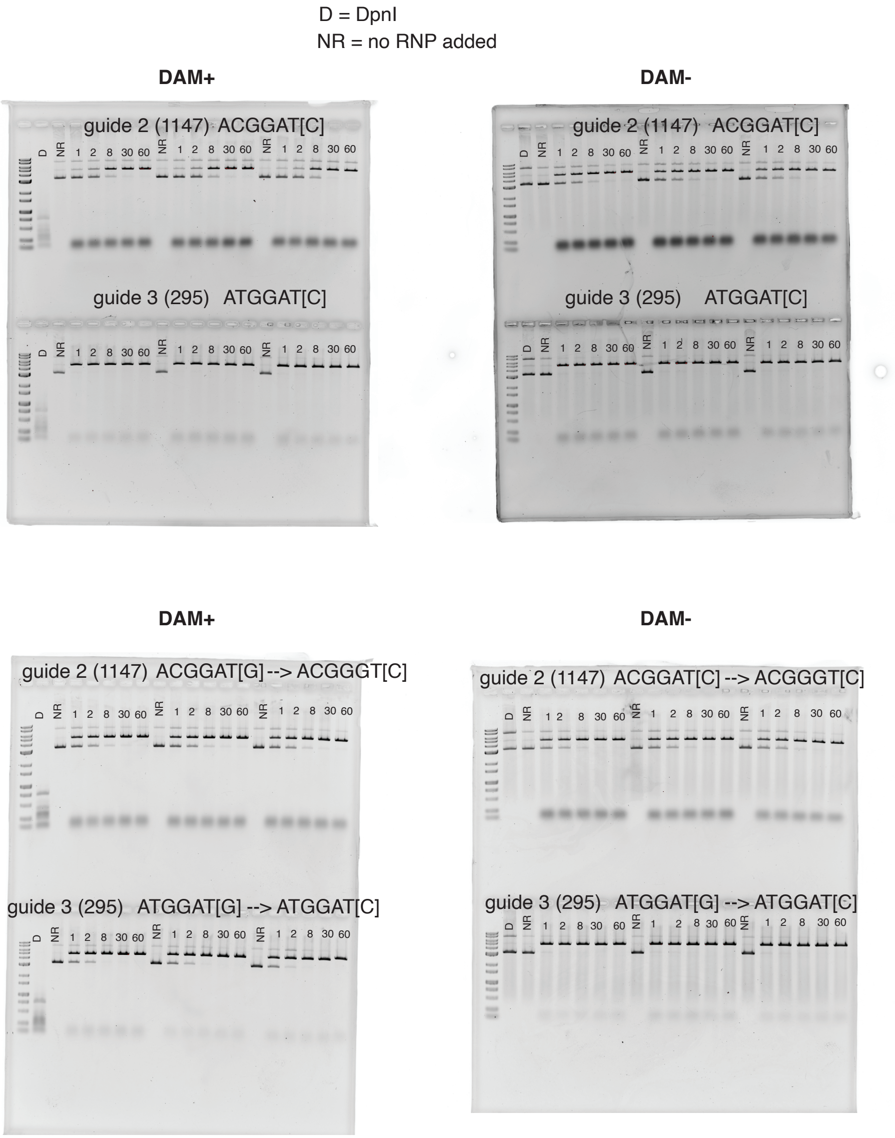

Figure S9: Agarose gel electrophoresis of cleavage reactions with SaCas9 and plasmid substrates isolated from DAM- or DAM+ *E. coli* strains using the indicated guide RNAs. The position of the guide RNA target site in the plasmid is indicated in a bracket. The bottom series of gels are for plasmid substrates with PAM site substitutions that ablate (top series) or create (bottom series) a GATC methylation site in the plasmid. D, DpnI digestion. NR, no RNP added.

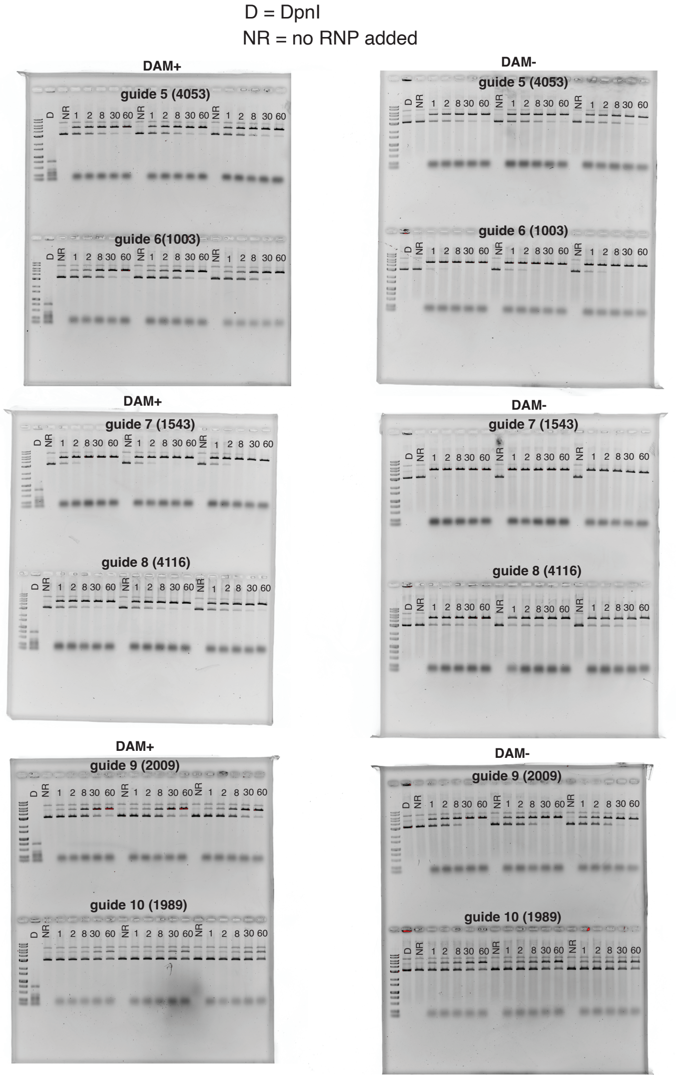

Figure S10: Agarose gel electrophoresis of cleavage reactions with SaCas9 and plasmid substrates isolated from DAM- or DAM+ *E. coli* strains using the indicated guide RNAs. The position of the guide RNA target site in the plasmid is indicated in a bracket. D, DpnI digestion. NR, no RNP added.

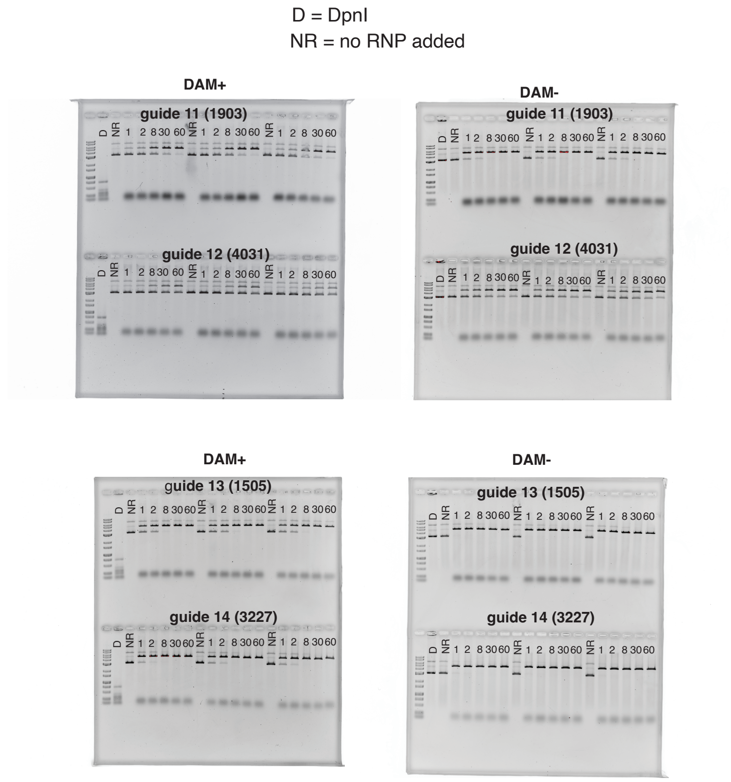

Figure S11: Agarose gel electrophoresis of cleavage reactions with SaCas9 and plasmid substrates isolated from DAM- or DAM+ *E. coli* strains using the indicated guide RNAs. The position of the guide RNA target site in the plasmid is indicated in a bracket. D, DpnI digestion. NR, no RNP added.

Supplementary Tables

Table S1: List of oligonucleotides

Name	Sequence (5′ to 3′)	Notes
DE5224	CCCTAAGAAATGAACTGGCAGC	Used in second strand synthesis reaction to make the oligo Pools of sgRNAs double stranded/Reverse primer to amplify *Citrobacter rodentium* sgRNA pool from Twist
DE5231	CCTGGTTCTTGGTCTCTCAC	Forward primer to amplify *Citrobacter rodentium* sgRNA pool from Twist
DE7766	ACACTCTTTCCCTACACGACGCTCTTCCGATCTNNNCAGTCGTTAAGAAGTGATAGAGATACTGAGCACG	Forward primer with Illumina adapter, 3 random nucleotides, 12-mer barcode (CAGTCGTTAAGA), loci-specific nts
DE7767	ACACTCTTTCCCTACACGACGCTCTTCCGATCTNNNCACTACGCTAGAAGTGATAGAGATACTGAGCACG	Forward primer with Illumina adapter, 3 random nucleotides, 12-mer barcode (CACTACGCTAGA), loci-specific nts
DE7768	ACACTCTTTCCCTACACGACGCTCTTCCGATCTNNNGCTCGAAGATTCAGTGATAGAGATACTGAGCACG	Forward primer with Illumina adapter, 3 random nucleotides, 12-mer barcode (GCTCGAAGATTC), loci-specific nts
DE7769	ACACTCTTTCCCTACACGACGCTCTTCCGATCTNNTGAACGTTGGATAGTGATAGAGATACTGAGCACG	Forward primer with Illumina adapter, 2 random nucleotides, 12-mer barcode (TGAACGTTGGAT), loci-specific nts
DE7770	ACACTCTTTCCCTACACGACGCTCTTCCGATCTNNATGGTTCACCCGAGTGATAGAGATACTGAGCACG	Forward primer with Illumina adapter, 2 random nucleotides, 12-mer barcode (ATGGTTCACCCG), loci-specific nts
DE7771	ACACTCTTTCCCTACACGACGCTCTTCCGATCTNNCGAGGGAAAGTCAGTGATAGAGATACTGAGCACG	Forward primer with Illumina adapter, 2 random nucleotides, 12-mer barcode (CGAGGGAAAGTC), loci-specific nts
DE7772	ACACTCTTTCCCTACACGACGCTCTTCCGATCTNTACTACGTGGCCAGTGATAGAGATACTGAGCACG	Forward primer with Illumina adapter, 1 random nucleotide, 12-mer barcode (TACTACGTGGCC), loci-specific nts
DE7773	ACACTCTTTCCCTACACGACGCTCTTCCGATCTNGTTCCTCCATTAAGTGATAGAGATACTGAGCACG	Forward primer with Illumina adapter, 1 random nucleotide, 12-mer barcode (GTTCCTCCATTA), loci-specific nts

DE7774	ACACTCTTTCCCTACACGACGCTCTTCCGATCTNACGATATGGTCAAGTGATAGAGATACTGAGCACG	Forward primer with Illumina adapter, 1 random nucleotide, 12-mer barcode (ACGATATGGTCA), loci-specific nts
DE7775	CGGTCTCGGCATTCCTGCTGAACCGCTCTTCCGATCTNNNACTCACAGGAATTTTAGTAGATTCTGTTTCCAGAGTAC	Reverse primer with Illumina adapter, 3 random nucleotides, 12-mer barcode (ACTCACAGGAAT), loci-specific nts
DE7776	CGGTCTCGGCATTCCTGCTGAACCGCTCTTCCGATCTNNGTAGGTGCTTACTTTAGTAGATTCTGTTTCCAGAGTAC	Reverse primer with Illumina adapter, 2 random nucleotides, 12-mer barcode (GTAGGTGCTTAC), loci-specific nts
DE7777	CGGTCTCGGCATTCCTGCTGAACCGCTCTTCCGATCTNCAGTCGTTAAGATTTAGTAGATTCTGTTTCCAGAGTAC	Reverse primer with Illumina adapter, 1 random nucleotide, 12-mer barcode (CAGTCGTTAAGA), loci-specific nts
DE7820	GGATCTAGGTGAAGATCCTTTTTGATAATC	Forward primer to amplify pTox+LacIq backbone
DE7821	GGTCTGACGCTCAGTGG	Reverse primer to amplify pTox+LacIq backbone
DE7830	GATTATCAAAAAGGATCTTCACCTAGATCCGGCTGGCCTGGAAGTTGGCATTTCGCTGCT	Forward primer to amplify *Citrobacter* gDNA fragment
DE7831	CATCCTGCATGTTCACCCACTGACGCAGCGTGGTCTGACAGTTACCAATGCTTAATCAGT	Reverse primer to amplify *Citrobacter* gDNA fragment
DE6018	AAAATCTCGCCAACAAGTTGACGAGATAAACACGGCATTTTGCCTTGTTTTAGTAGATTCTGTTTCCAGAGTACTAAAAC	Universal scaffold primer for SaCas9 sgRNAs
DE8170	aagcTAATACGACTCACTATATGGTTGGCTTACGTTTTTTCGGTTTTAGTACTCTGGAAACAG	$sgRNA_{1447}$ for pTox-KatG
DE8171	aagcTAATACGACTCACTATAAGGCCGCGATTAAATTCCAACGTTTTAGTACTCTGGAAACAG	$sgRNA_{295}$ for pTox-KatG
DE8802	aagcTAATACGACTCACTATAGAGCAGATTACGCGCAGAAAAAGTTTTAGTACTCTGGAAACAG	$sgRNA_{4053}$ for pTox-KatG
DE8803	aagcTAATACGACTCACTATAGATCTACGTTAACCCGGAAGGGGTTTTAGTACTCTGGAAACAG	$sgRNA_{1003}$ for pTox-KatG
DE8804	aagcTAATACGACTCACTATAGCAAAGGCTTCATTAAAGGCCTGTTTTAGTACTCTGGAAACAG	$sgRNA_{1543}$ for pTox-KatG
DE8805	aagcTAATACGACTCACTATAGCAGACCCCGTAGAAAAGATCAGTTTTAGTACTCTGGAAACAG	$sgRNA_{4116}$ for pTox-KatG
DE8806	aagcTAATACGACTCACTATAGCAGCAGACGTGCACTGGCCAGGTTTTAGTACTCTGGAAACAG	$sgRNA_{2860}$ for pTox-KatG

DE8807	aagcTAATACGACTCACTATAGCCGGGCCGGGTGGATGCGCGTGTTTTAGTACTCTGGAAACAG	$sgRNA_{1989}$ for pTox-KatG
DE8808	aagcTAATACGACTCACTATAGCGAGGCTTTATTCGTCGTTTTGTTTTAGTACTCTGGAAACAG	$sgRNA_{1903}$ for pTox-KatG
DE8809	aagcTAATACGACTCACTATAGCTACCAGCGGTGGTTTGTTTGGTTTTAGTACTCTGGAAACAG	$sgRNA_{4031}$ for pTox-KatG
DE8810	aagcTAATACGACTCACTATAGGACGGGAAATCTTCTCGAACTGTTTTAGTACTCTGGAAACAG	$sgRNA_{1505}$ for pTox-KatG
DE8811	aagcTAATACGACTCACTATAGGCACACTGGCGGCCGTTACTAGTTTTAGTACTCTGGAAACAG	$sgRNA_{3227}$ for pTox-KatG
DE8259	6-FAM/CGTTCGATCCGTCGAAAAAACGTAAGCCAACCATGCTGGTCACCG	Complement of DE8260, synthetic methyl cytosine substrate
DE8260	6-FAM/CGGTGACCAGCATGGTTGGCTTACGTTTTTTCGACGGAT/iMe-dC/GAACG	Synthetic SaCas9 substrate with 5mC modification in PAM[+1] sequence
DE9076	6-FAM/ACGCGCCGGACATCATCCCGGACCCGTTCGATCCGTCGAAAAAACGTAAGCCAACCATGTGGTCACCG	Ummethylated synthetic substrate
DE9078	6-FAM/CGGTGACCAGCATGGTTGGCTTACGTTTTTTCGACGGATCGAACGGGTCCGGGATGATGTCCGGCGCGT	Non-methylated synthetic substrate
DE9077	6-FAM/CGGTGACCAGCATGGTTGGCTTACGTTTTTTCGACGGAT/iMeth-dC/GAACGGGTCCGGGATGATGTCCGGCGCGT	Synthetic substrate with with 5mC modification in PAM[+1] sequence
DE9116	6-FAM/ACGCGCCGGACATCATCCCGGACCCGTTCGAT/iMeth-dC/CGTCGAAAAAACGTAAGCCAACCATGCTGGTCACCG	Synthetic substrate with with 5mC modification in PAM[+1] sequence

		DAM(+)		DAM(-)		
**sgRNA:PAM[+1]**	**pTox position**	k_{obs}	**std.dev**	k_{obs}	**std.dev**	**DAM(-) / DAM(+)**
2:ACGGAT[C]	1447	0.19	0.03	0.63	0.22	3.32
2:ACGGGT[C]	1447	0.70	0.18	0.77	0.18	1.11
3:ATGGAT[G]	295	4.63	0.78	3.76	0.94	0.81
3:ATGGAT[C]	295	1.11	0.11	3.38	0.78	3.06
5:AAGGAT[C]	4053	0.41	0.19	0.51	0.33	1.24
6:CCGGAT[C]	1003	0.10	0.03	1.66	0.22	16.86
7:GCGGAT[C]	1543	1.24	0.04	3.66	0.45	2.96
8:AAGGAT[C]	4116	0.68	0.20	0.86	0.41	1.26
9:GGGGAT[C]	2860	0.04	0.01	0.18	0.06	4.35
10:CAGGAT[C]	1989	0.01	0.00	0.01	0.00	1.75
11:CTGGAT[C]	1903	0.08	0.03	1.31	0.12	15.72
12:CCGGAT[C]	4031	0.01	0.00	0.03	0.03	3.20
13:CCGGAT[C]	1505	0.62	0.04	5.15	1.45	8.24
14:AAGGAT[C]	3227	1.33	0.12	3.69	0.64	2.77

Table S6: Observed reaction rates (min^{-1}) for sgRNAs targeting pTox-KatG at the indicated position. The PAM[+1] sequence for each target site is indicated, and substitutions to the sequence are indicated by underlined nucleotides.
